# Supplementary material for: A Pilot Study of the Clinical Effectiveness of a Single Intra-Articular Injection of Stanozolol in Canines with Knee Degenerative Joint Disease and Its Correlation with Serum Interleukin-1β Levels
Source: Animals (Basel). 2024 Apr 30;14(9):1351. doi: 10.3390/ani14091351 (PMC11082967; doi:10.3390/ani14091351)
Supplement: Supplementary file 1 [file animals-14-01351-s001.zip › animals-2933761-supplementary.pdf]

Table S1. Form used for recording during the clinical trial, as well as the study variables and owners' opinions.

**Evaluation by Clinicians**													
**types of lameness**													
T0	absent	0	starts cold	1	starts warm	2	starts cold,worsens with exercise	3	permanent	4			
T2	absent	0	starts cold	1	starts warm	2	starts cold,worsens with exercise	3	permanent	4			
T2	absent	0	starts cold	1	starts warm	2	starts cold,worsens with exercise	3	permanent	4			
T3	absent	0	starts cold	1	starts warm	2	starts cold,worsens with exercise	3	permanent	4			
**Describing the pain on manipulation at this moment**													
T0	absent	0	1	2	3	4	5	6	7	8	9	intense pain	10
T2	absent	0	1	2	3	4	5	6	7	8	9	intense pain	10
T2	absent	0	1	2	3	4	5	6	7	8	9	intense pain	10
T3	absent	0	1	2	3	4	5	6	7	8	9	intense pain	10
**To what extent does the pain interfere with the patient's ability to stand up after lying down**													
T0	Doesn´t interfere	0	1	2	3	4	5	6	7	8	9	completely interferes	10
T2	Doesn´t interfere	0	1	2	3	4	5	6	7	8	9	completely interferes	10
T2	Doesn´t interfere	0	1	2	3	4	5	6	7	8	9	completely interferes	10
T3	Doesn´t interfere	0	1	2	3	4	5	6	7	8	9	completely interferes	10
**To what extent does the pain interfere with the patient's ability to walk**													
T0	Doesn´t interfere	0	1	2	3	4	5	6	7	8	9	completely interferes	10
T2	Doesn´t interfere	0	1	2	3	4	5	6	7	8	9	completely interferes	10
T2	Doesn´t interfere	0	1	2	3	4	5	6	7	8	9	completely interferes	10
T3	Doesn´t interfere	0	1	2	3	4	5	6	7	8	9	completely interferes	10
**To what extent does the pain interfere with the patient's ability to run**													
T0	Doesn´t interfere	0	1	2	3	4	5	6	7	8	9	completely interferes	10
T2	Doesn´t interfere	0	1	2	3	4	5	6	7	8	9	completely interferes	10
T2	Doesn´t interfere	0	1	2	3	4	5	6	7	8	9	completely interferes	10
T3	Doesn´t interfere	0	1	2	3	4	5	6	7	8	9	completely interferes	10
**How would you classify the current quality of life of the patient**													
T0	Poor	fair	good	very good	excellent								
T2	Poor	fair	good	very good	excellent								
T2	Poor	fair	good	very good	excellent								
T3	Poor	fair	good	very good	excellent								
**Evaluation by Tutors**													
**What best describes the major pain level experienced by your pet during the last 7 days**													
T0	absent	0	1	2	3	4	5	6	7	8	9	intense pain	10
T2	absent	0	1	2	3	4	5	6	7	8	9	intense pain	10
T2	absent	0	1	2	3	4	5	6	7	8	9	intense pain	10
T3	absent	0	1	2	3	4	5	6	7	8	9	intense pain	10
**What best describes the minor pain level experienced by your pet during the last 7 day**													
T0	absent	0	1	2	3	4	5	6	7	8	9	intense pain	10
T2	absent	0	1	2	3	4	5	6	7	8	9	intense pain	10
T2	absent	0	1	2	3	4	5	6	7	8	9	intense pain	10
T3	absent	0	1	2	3	4	5	6	7	8	9	intense pain	10
**What best describes the average level of pain experienced during the last 7 days**													
T0	absent	0	1	2	3	4	5	6	7	8	9	intense pain	10
T2	absent	0	1	2	3	4	5	6	7	8	9	intense pain	10
T2	absent	0	1	2	3	4	5	6	7	8	9	intense pain	10
T3	absent	0	1	2	3	4	5	6	7	8	9	intense pain	10

**Describing the pain on manipulation at this moment**													
T0	absent	0	1	2	3	4	5	6	7	8	9	intense pain	10
T2	absent	0	1	2	3	4	5	6	7	8	9	intense pain	10
T2	absent	0	1	2	3	4	5	6	7	8	9	intense pain	10
T3	absent	0	1	2	3	4	5	6	7	8	9	intense pain	10
**Function description**													
**Describe how pain has interfered with overall activity in the last 7 days**													
T0	Doesn´t interfere	0	1	2	3	4	5	6	7	8	9	completely interferes	10
T2	Doesn´t interfere	0	1	2	3	4	5	6	7	8	9	completely interferes	10
T2	Doesn´t interfere	0	1	2	3	4	5	6	7	8	9	completely interferes	10
T3	Doesn´t interfere	0	1	2	3	4	5	6	7	8	9	completely interferes	10
**Describe how pain has interfered with quality of life in the last 7 days**													
T0	Doesn´t interfere	0	1	2	3	4	5	6	7	8	9	completely interferes	10
T2	Doesn´t interfere	0	1	2	3	4	5	6	7	8	9	completely interferes	10
T2	Doesn´t interfere	0	1	2	3	4	5	6	7	8	9	completely interferes	10
T3	Doesn´t interfere	0	1	2	3	4	5	6	7	8	9	completely interferes	10
**Describe how pain has interfered with the ability to stand up after lying down in the last 7 days**													
T0	Doesn´t interfere	0	1	2	3	4	5	6	7	8	9	completely interferes	10
T2	Doesn´t interfere	0	1	2	3	4	5	6	7	8	9	completely interferes	10
T2	Doesn´t interfere	0	1	2	3	4	5	6	7	8	9	completely interferes	10
T3	Doesn´t interfere	0	1	2	3	4	5	6	7	8	9	completely interferes	10
**Describe how pain has interfered with the ability to walk in the last 7 days**													
T0	Doesn´t interfere	0	1	2	3	4	5	6	7	8	9	completely interferes	10
T2	Doesn´t interfere	0	1	2	3	4	5	6	7	8	9	completely interferes	10
T2	Doesn´t interfere	0	1	2	3	4	5	6	7	8	9	completely interferes	10
T3	Doesn´t interfere	0	1	2	3	4	5	6	7	8	9	completely interferes	10
**Describe how pain has interfered with the ability to run in the last 7 days**													
T0	Doesn´t interfere	0	1	2	3	4	5	6	7	8	9	completely interferes	10
T2	Doesn´t interfere	0	1	2	3	4	5	6	7	8	9	completely interferes	10
T2	Doesn´t interfere	0	1	2	3	4	5	6	7	8	9	completely interferes	10
T3	Doesn´t interfere	0	1	2	3	4	5	6	7	8	9	completely interferes	10
**Describe how pain has interfered with the ability to climb stairs, sidewalks, steps, ... in the last 7 days**													
T0	Doesn´t interfere	0	1	2	3	4	5	6	7	8	9	completely interferes	10
T2	Doesn´t interfere	0	1	2	3	4	5	6	7	8	9	completely interferes	10
T2	Doesn´t interfere	0	1	2	3	4	5	6	7	8	9	completely interferes	10
T3	Doesn´t interfere	0	1	2	3	4	5	6	7	8	9	completely interferes	10

**Overall appreciation**					
**which best describes the level of quality of life of your dog in the last 7 days**					
T0	Poor	fair	good	very good	excellent
T2	Poor	fair	good	very good	excellent
T2	Poor	fair	good	very good	excellent
T3	Poor	fair	good	very good	excellent
